# Investigation of vibration’s effect on driver in optimal motion cueing algorithm

**DOI:** 10.1371/journal.pone.0290705

**Published:** 2023-11-30

**Authors:** Hazoor Ahmad, Muhammad Tariq, Awais Yasin, Sohail Razzaq, Muhammad Ahmad Chaudhry, Inam Ul Hasan Shaikh, Ahsan Ali, Saeed Mian Qaisar, Jamshed Iqbal

**Affiliations:** 1 Department of Electrical Engineering, Information Technology University (ITU), Lahore, Pakistan; 2 Alternate Energy Research & Innovation Lab (AERIL), Al-Khwarizmi Institute of Computer Science, University of Engineering and Technology, Lahore, Pakistan; 3 Computer Engineering Department, National University of Technology (NUTech), Islamabad, Pakistan; 4 Faculty of Information Technology, Majan University College, Muscat, Sultanate of Oman; 5 Department of Electrical Engineering, University of Engineering and Technology (UET), Taxila, Pakistan; 6 Electrical and Computer Engineering Department, Effat University, Jeddah, Saudi Arabia; 7 CESI LINEACT, Lyon, France; 8 School of Computer Science, Faculty of Science and Engineering, University of Hull, Hull, United Kingdom; Universiti Sains Malaysia, MALAYSIA

## Abstract

The increased sensation error between the surroundings and the driver is a major problem in driving simulators, resulting in unrealistic motion cues. Intelligent control schemes have to be developed to provide realistic motion cues to the driver. The driver’s body model incorporates the effects of vibrations on the driver’s health, comfort, perception, and motion sickness, and most of the current research on motion cueing has not considered these factors. This article proposes a novel optimal motion cueing algorithm that utilizes the driver’s body model in conjunction with the driver’s perception model to minimize the sensation error. Moreover, this article employs H∞ control in place of the linear quadratic regulator to optimize the quadratic cost function of sensation error. As compared to state of the art, we achieve decreased sensation error in terms of small root-mean-square difference (70%, 61%, and 84% decrease in case of longitudinal acceleration, lateral acceleration, and yaw velocity, respectively) and improved coefficient of cross-correlation (3% and 1% increase in case of longitudinal and lateral acceleration, respectively).

## Introduction

Evaluation of Virtual Reality in driving and on-road field studies have shown encouraging resemblances [1, 2]. However, when compared to natural world driving, questions are raised on the validity of driving simulators. It will not be easy to perceive a driver’s behavior in such an environment. Driving simulators are considered very important to improve road safety through proper indoor training and improved vehicle design [3]. Their use is crucial when the number of vehicles is considerably large, and road structure is also not very appropriate. Research has shown that a virtually trained driver saves money, time, life, and material. Nowadays, driving simulators are used not only as training simulators but also as entertainment simulators. Researchers have evaluated drivers’ behavior, performance, and attention. In the automobile industry, they are used for optimal and risk-free vehicle design, resulting in reduced vehicle cost and increased safety. We can have efficient hardware implementations of driving simulators using energy efficiency techniques, such as SuperSlash [4], which is used for energy efficiency of artificial intelligence (AI) based models.

The motion cueing algorithm (MCA) converts linear accelerations and angular velocities of a virtual vehicle into movements of a driving simulator. One of the first methods to transform these accelerations and velocities was the classical MCA proposed by Conrad et al. [5]. Classical MCA is a linear cueing algorithm that is simple and easily tunable and has been widely used in different types of simulators [1, 6]. Classical motion cueing has a short processing time and stable performance [7]. However, classical MCA has many drawbacks. For example, it needs a regulation procedure that emphasizes the worst-case situation resulting in conventional motion with deprived workspace usage, hence not being flexible in all situations. Moreover, it completely ignores the human vestibular system [8] and may generate false cues, distort the signal, and lead to wrong motion signals due to filter characteristics [9]. Also, a low-pass filter leads to phase delay [10]. The coordinated adaptive washout algorithm has been applied to flight simulators in [11]. The authors replaced the tilt coordination block of the classical washout filter with an adaptive filter to avoid residual motion base displacements. In [12], adaptive motion cueing using fuzzy-based tilt coordination has been proposed. Due to many other possibilities of tilt coordination [13], the study of this algorithm is just at its initial stages.

An optimal MCA was proposed ignoring the vestibular system [14]. Optimal MCA has some drawbacks, but it generally reduces the sensation error between the virtual surrounding and real drivers. However, the results are not satisfactory to a real human perception due to physical limitations and an optimal washout filter needs to be applied to a larger workspace [15]. Getting realistic motion cues on a six-degree-of-freedom Stewart platform compared to the actual vehicle is still a problem. In some cases, the motion system has to be effectively turned off to avoid improper motion cues. A robust optimal MCA based on the linear quadratic regulator (LQR) method and the genetic algorithm (GA) has been studied in [16]. The driver feedback has been used in [17] to characterize desirable features of race cars; then the numerical optimal control (NOC) was used to decide platform motions. NOC is a useful open-loop method for analyzing performance constraints of the driver in loop strategies [17]. Other methods, including LQR-based optimal control and GA-based optimal control [16], exhibit better performance in improving the sensation output, producing realistic motion cues but cannot enhance workspace usage due to several constraints. The LQR-based method improves the workspace, but it has more sensation error and the GA-based method decreases error but with poor workspace usage [16]. Recently, many methods have introduced neural networks and computational intelligence-based motion cueing [18, 19]; however, these techniques are computationally complex.

This research utilizes H∞-based optimal MCA, which shows superior performance compared to the state-of-the-art methods. This article has the following main contributions:

1. A novel optimal MCA is proposed that utilizes the driver’s body model together with the driver’s perception model.
2. The LQR is replaced with an H∞-based controller to optimize the quadratic cost function of sensation error.

A significant advantage of using H∞-based MCA is that it can be used for any driving simulator without parameter tuning. We need to adjust the physical constraints of the new system and do not need to care about “trial and error” for parameter tuning.

The article is organized as follows: the Background section comprises the math behind the Stewart platform and its kinematics; the Proposed Methodology section includes the application of proposed optimal MCA on the Stewart platform; the Results and Discussion section compares different motion cueing techniques. Finally, the Conclusion section concludes the article by discussing the improvements made by the proposed methodology.

## Background

**Fig 1:**
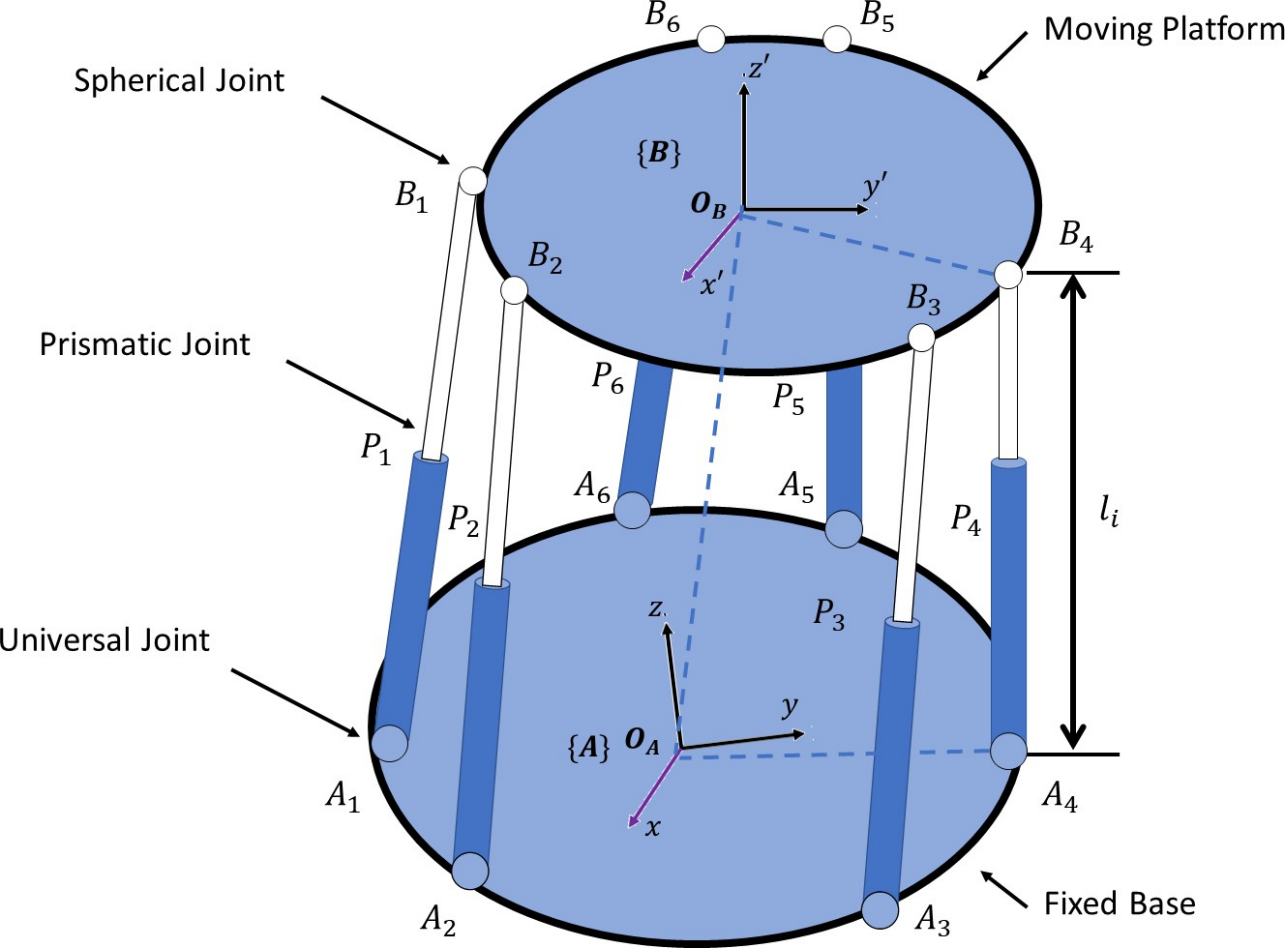
A generic Stewart platform with basic frames and parameters.

In this section, we will discuss the mathematics behind the Stewart platform along with its kinematics. Fig 1 refers to the schematics of a generic Stewart platform. There are two parts to every limb length (*l*_*i*_) connected by a prismatic joint (*P*_*i*_). This limb’s upper and lower sides are connected to the upper and lower platforms with spherical (or universal) joints. In this robot, six identical hydraulic actuators can be actuated to obtain six-degree-of-freedom motions of the moving platform. Six universal joints at the base platform of the Stewart platform are referred to as *A*_*i*_, where *i* = 1, 2, … 6. Similarly, six spherical joints at the moving platform of the Stewart platform are referred to as *B*_*i*_, where *i* = 1, 2, … 6. The prismatic joints between fixed and moving platforms are referred to as *P*_*i*_, where *i* = 1, 2, … 6. The limb length of every kinematic chain of Stewart platform is referred to as li , where *i* = 1, 2, … 6. Two frames *O*_*A*_ and *O*_*B*_ are attached to the fixed and moving platforms, respectively.

### Inverse kinematics

Inverse kinematics deals with the determination of limb lengths when the position and orientation of the moving platform are known [20–22]. In the case of a parallel robot, like a Stewart platform, inverse kinematics is easy [23]. The position AP of the moving platform and orientation $\hat{s}$ are given, and limb lengths *l*_*i*_ s are to be determined. The position is given as follows:

$${}^{A}P={\left[\begin{array}{c} p_{x}\;\;\;p_{y}\;\;\;p_{z} \end{array}\right]}^{T}.$$ (1)

In the case of screw axis representation, the orientation of the platform is given as follows:

$$\left\{\hat{\text{s}}=\left[\begin{array}{c} s_{x}\;\;\;s_{y}\;\;\;s_{z} \end{array}\right],\theta \right\}.$$ (2)

In the case of rotation matrix representation, the orientation of the platform is given as follows:

$${}_{B}^{A}R=\left[\begin{array}{c} u_{x}\;\;\;v_{x}\;\;\;w_{x}\\ u_{y}\;\;\;v_{y}\;\;\;w_{y}\\ u_{z}\;\;\;v_{z}\;\;\;w_{z} \end{array}\right].$$ (3)

Using the loop closure equation for each limb on the geometry of Fig 1,

lis^iA=PA+bAi−aAi, (4)

where ${}_{B}^{A}b_{i}{=}_{B}^{A}R{\;}^{B}b_{i}$.

Eliminating $\hat{\text{s}}$ from Eq (4),

$$\begin{array}{c} l_{i}=\left[{}_{B}^{A}P^{T}{}^{A}P{+}^{B}b_{i}^{T}{}_{i}^{B}b{+}^{A}a_{i}^{T}{}^{A}a_{i}-2^{A}P^{T}{}^{A}a_{i}+2^{A}P^{T}\left[{}_{B}^{A}R^{B}b_{i}\right]\right.\\ {\left.-2{\left[{}_{B}^{A}R^{B}b_{i}\right]}^{T}{}^{A}a_{i}\right]}^{1/2}\cdot \end{array}$$ (5)

The procedure in [17] will be followed to find actuator velocity. Taking the derivative of Eq (5) and simplifying for angular velocity, we get

$${}_{B}^{A}\dot{P}={\dot{l}}_{i}{\;}_{i}^{A}\hat{\text{s}}{-}_{i}^{A}\dot{b}{+}_{i}^{A}\dot{a}.$$ (6)

### Forward kinematics

Forward kinematics is the method of determining the position and orientation of moving platforms when limb lengths of the Stewart platform are known. As shown in Fig 1, if vector L of limb lengths *l*_*i*_, where *i* = 1,2,⋯,6, is known, then the problem is to determine position ^*A*^*P* and orientation ${}_{B}^{A}R$.

The optimal solution method having many iterations needs high-speed computation due to the heavy computational burden [24]. A good initial guess has to be made to establish a rapid and converging solution. Forward kinematics can be obtained using nonlinear least-squares algorithms based on Levenberg–Marquardt or trust-region-reflective methods [23]. The trust-region-reflective method is based on the interior-reflective Newton method proposed in [25]. The Levenberg–Marquardt method has been proposed in [26, 27]. To apply one of these algorithms, the first step will be to merge Eqs (1) and (2):

$$t=\left[\begin{array}{c} p_{x}\;\;\;p_{y}\;\;\;p_{z}\;\;\;s_{x}\;\;\;s_{y}\;\;\;s_{z}\;\;\;\theta \end{array}\right]$$ (7)

or, equivalently,

$$t=\left[\begin{array}{c} t_{1}\;\;\;t_{2}\;\;\;t_{3}\;\;\;t_{4}\;\;\;t_{5}\;\;\;t_{6}\;\;\;t_{7} \end{array}\right].$$ (8)

Position ^*A*^*P* and orientation ${}_{B}^{A}R$ will be determined as follows:

$$P(t)=\left[\begin{array}{c} t_{1}\;\;\;t_{2}\;\;\;t_{3} \end{array}\right]$$ (9)

and

$$R(t)=\left[\begin{array}{ccc} t_{4}^{2}vt_{7}+ct_{7} & t_{4}t_{5}vt_{7}-t_{6}st_{7} & t_{4}t_{6}vt_{7}+t_{5}st_{7}\\ t_{5}t_{4}vt_{7}+t_{6}st_{7} & t_{5}^{2}vt_{7}+ct_{7} & t_{5}t_{6}vt_{7}-t_{4}st_{7}\\ t_{6}t_{4}vt_{7}-t_{5}st_{7} & t_{6}t_{5}vt_{7}+t_{4}st_{7} & t_{6}^{2}vt_{7}+ct_{7} \end{array}\right],$$ (10)

where *vt*_7_ = (1-cos (*t*_7_)), *st*_7_ = sin (*t*_7_) and *ct*_7_ = cos (t7).


$$s(t)=\left[\begin{array}{c} t_{4}\;\;\;t_{5}\;\;\;t_{6}\;\;t_{7} \end{array}\right].$$ (11)

From Fig 1, the moving platform has points *B*_*i*_’s and the base platform has points *A*_*i*_’s and their vectors with respect to the base are *b*_*i*_’s and *a*_*i*_’s, respectively. The following set of nonlinear equations can be solved for the forward kinematics:

$$F_{i}(t)=-l_{i}^{2}+{\left[P(t)+R(t)b_{i}-a_{i}\right]}^{T}\left[P(t)+R(t)b_{i}-a_{i}\right]$$ (12)

and

$$F_{7}(t)=\sum_{k=4}^{6}t_{k}^{2}-1.$$ (13)

Optimization methods can be employed here to solve the above seven nonlinear equations (Eqs (12) and (13)). In case of least-squares optimization, minimize $f(t)=\frac{1}{2}\sum_{i}F_{i}{\left(t\right)}^{2}$, where *t* ∈ ℛ.

**Fig 2:**
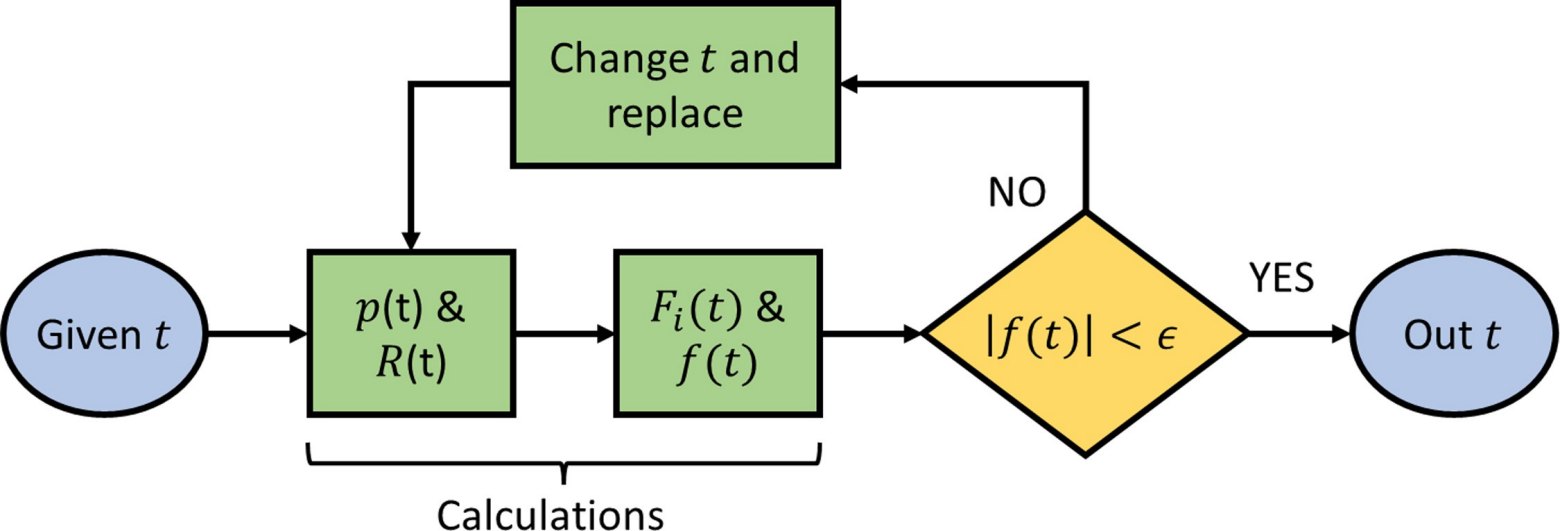
Iterative solution of forward kinematics.

The solution of forward kinematics is demonstrated as a flowchart in Fig 2. In the first flowchart process, Eqs 9 and 10 are used for the calculation of *p* and *R*, respectively. Then, block *F*_*i*_ is evaluated using Eq (12). Now, *f*(*t*) is determined as demonstrated above. The conditional block checks if this value is less than required accuracy *ε* or not. In case *f*(*t*) is less than *ε*, the function iteratively calculates the new value of *t* and sends it back to calculate *p* and *R*, to recalculate the new values of *p* and *R* corresponding to the new value of *t*.

**Fig 3:**
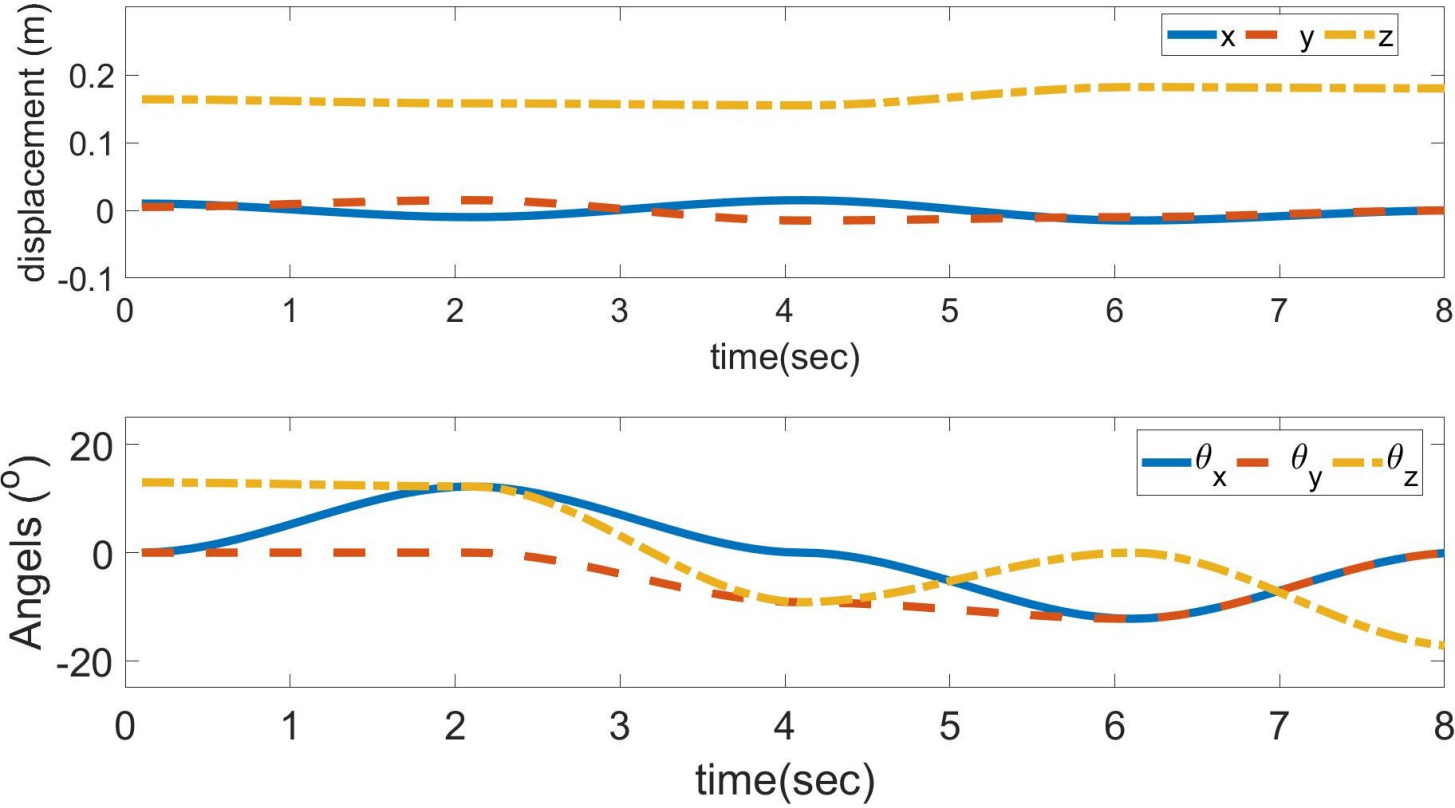
Supposed trajectories of position and orientation.

**Fig 4:**
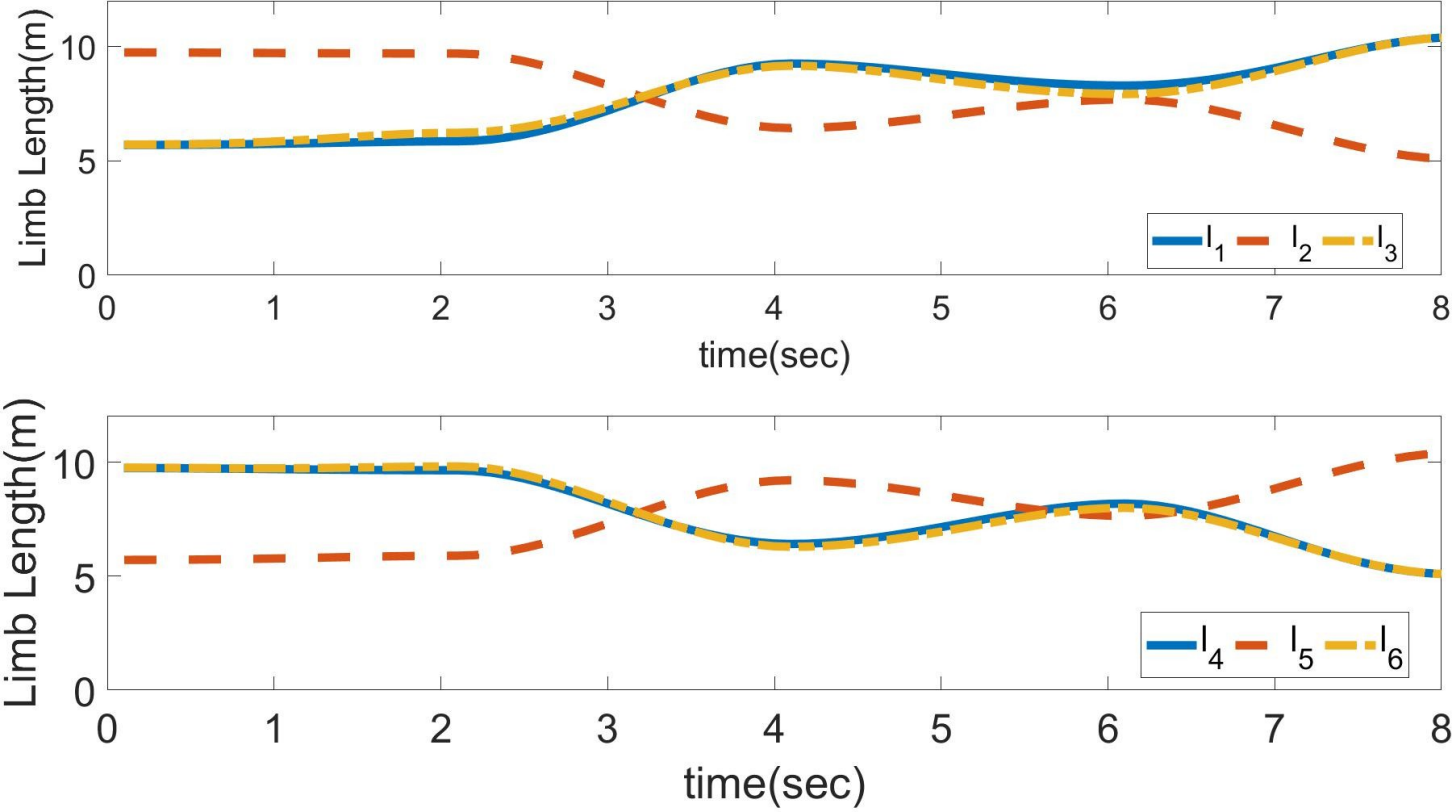
Calculated values of limb lengths.

The input trajectories of position and orientation are shown in Fig 3. The calculated limb lengths of the Stewart platform are obtained using inverse kinematics, as shown in Fig 4. The method of forward kinematics using the Levenberg–Marquardt method is applied to get values of position and orientation back from values of limb lengths of Fig 4. These values are compared with the previous position and orientation with negligible error.

### Motion cueing algorithm

Motion cueing refers to the approaches through which the environment of a driving simulator is made to feel like a real environment. These approaches are known as cues and are classified as acoustic, visual, and motion cues. A driving simulator of any class must contain an efficient visual system. Moreover, most visual systems are accompanied by acoustic systems. Motion simulators are required to provide motion cues sensed by vestibular, body, and perception system models. In the real environment, motion system parameter values exceed the limits of the driving simulator motion system. Therefore, it is essential to convert those parameters. The methods applied to perform this conversion are known as MCAs, motion drive algorithms, or washout filters. These algorithms aim to reproduce the motion cues in a realistic way, ensuring the usage of maximum workspace. The first was classical motion cueing built in [5] for flight simulators. Naturally, the requirements for a driving simulator are somewhat different; nonetheless, the classical algorithm can be adapted with some modifications.

## Proposed methodology

Optimal MCA for vehicle driving simulators that incorporate driver’s body and perception models is proposed so as to include human body vibrations [15, 28–30]. Human vibrations are of three types: the mass-spring-damper model [31], the model from the finite element method [32], and the transfer function model [14]. Methods for measuring periodic, random, and transient whole-body vibrations have been discussed in [28].

**Fig 5:**
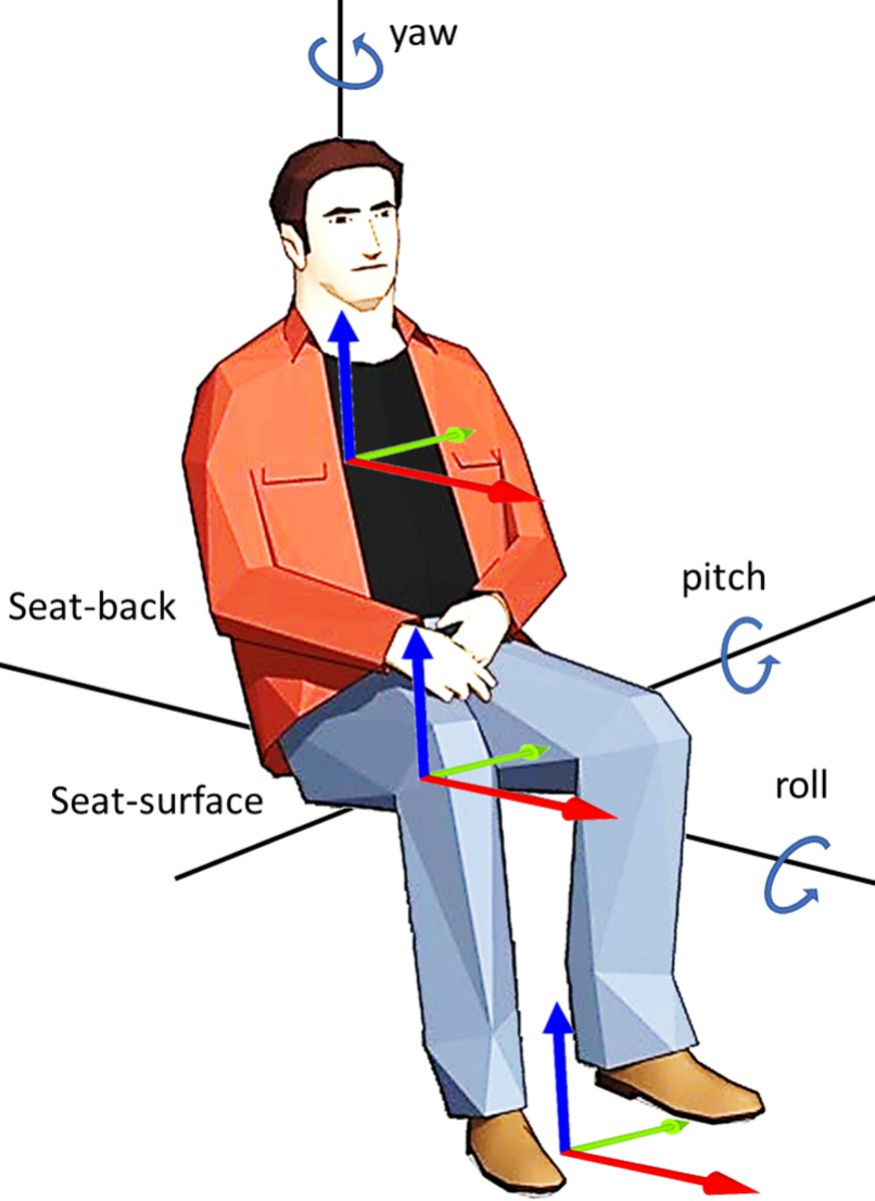
Basicentric coordinate system of the driver body (seated position).

Fig 5 shows the basicentric coordinate system of a human body commonly used to illustrate human body vibrations. The driver’s body model is given by Eqs (14–19) as a transfer function that contains a band-limiting filter GBL in Eq (18). This filter contains high-pass filter *G*_*HP*_ and low-pass filter *G*_*LP*_:

$$G_{HP}=\frac{s^{2}}{s^{2}+\alpha_{1}\sqrt{2}s+\alpha_{1}^{2}},$$ (14)

$$G_{LP}=\frac{1}{s^{2}+\alpha_{2}\sqrt{2}s+\alpha_{2}^{2}},$$ (15)

$$G_{BL}=\;G_{HP}\times \;G_{LP},$$ (16)

$$G_{T}=\frac{1+\frac{s}{\alpha_{3}}}{1+\frac{s}{H_{4}\alpha_{4}}+{\left(\frac{s}{\alpha_{4}}\right)}^{2}},$$ (17)

$$G_{s}=\frac{1+\frac{s}{H_{5}\alpha_{5}}+{\left(\frac{s}{\alpha_{5}}\right)}^{2}}{1+\frac{s}{H_{6}\alpha_{6}}+{\left(\frac{s}{\alpha_{6}}\right)}^{2}}\times {\left(\frac{\alpha_{5}}{\alpha_{6}}\right)}^{2},$$ (18)

$$G=\;G_{BL}\times \;G_{T}\;\times \;\;G_{s},$$ (19)

where *a*_*i*_, *i* = 1, 2, 3, … ,6 are the frequency weighted acceleration values and *H*_*i*_, *i* = 1, 2, 3, … ,6 are the resonant quality factors. These factors evaluate the overall frequency weighting of the transfer function. The values of these variables are obtained from [28].

The driver’s perception model comprises vestibular and otolith organs. These organs are responsible for the perception of rotational velocity and specific force. Specific force is the difference between linear acceleration and gravity. Two transfer functions are shown in Eqs (20) and (21). The first transfer function is the ratio of the vestibular organ to that of rotational velocity:

$$G_{\Omega }=\frac{L_{L}L_{A}s^{2}}{\left(1+L_{L}s\right)\left(1+L_{S}s\right)\left(1+L_{A}s\right)}.$$ (20)

The second transfer function is the ratio of the otolith organ to the specific force given as follows:

$$G_{\rho }=\frac{k\left(1+\tau_{A}s\right)}{\left(1+\tau_{s}s\right)\left(1+\tau_{L}s\right)},$$ (21)

where *L*_*L*_, *L*_*A*_, and *L*_*S*_ are the weighting factors from vestibular organ and *τ*_*L*_, *τ*_*A*_, and *τ*_*S*_ are weighting factors from the otolith organ.

### Problem statement

**Fig 6:**
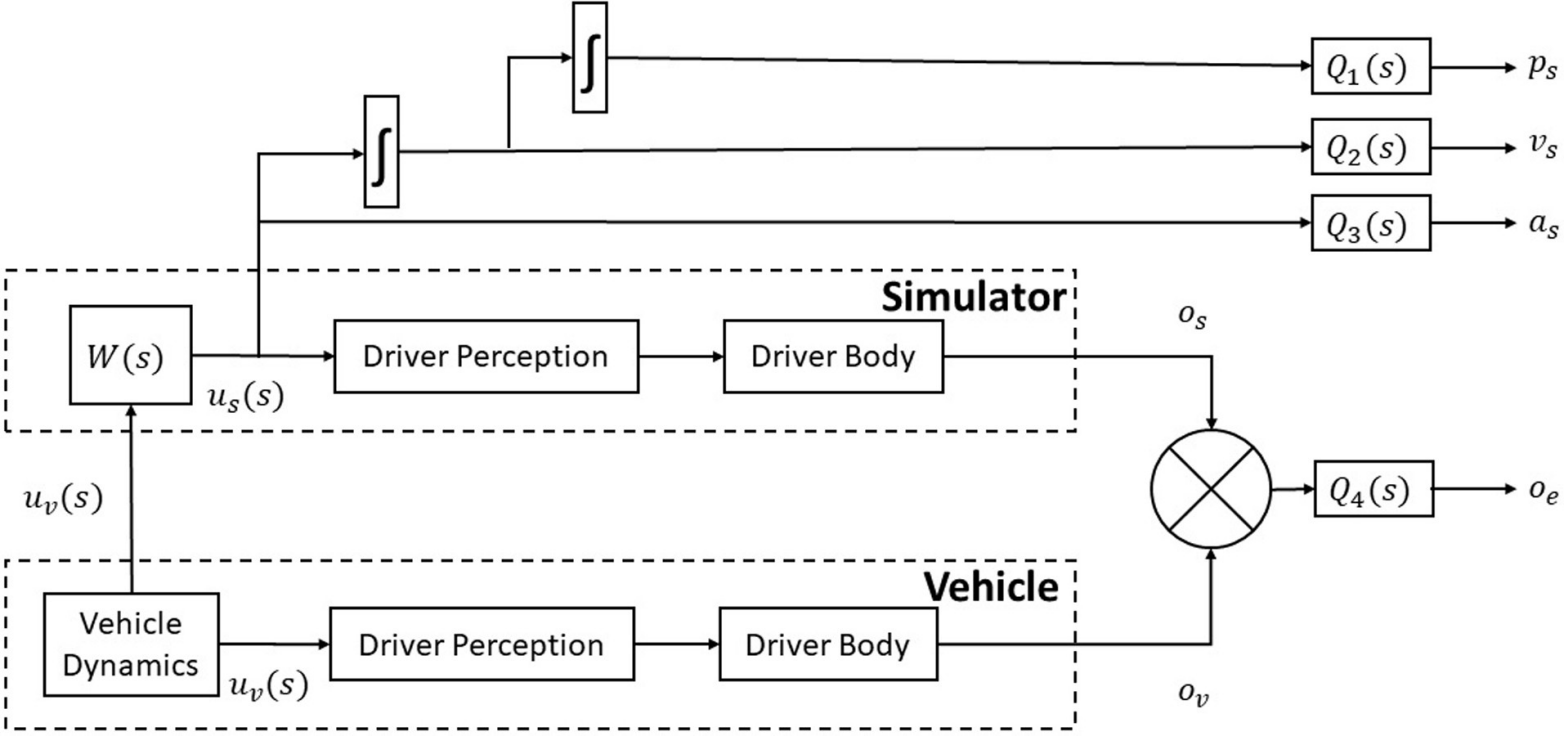
Proposed optimal cueing framework.

The block diagram of a driving simulator, including an optimal motion cueing block *W*(*s*), is shown in Fig 6.

The input to this block is *u*_*v*_(*s*) and its output is *u*_*s*_(*s*), which serves as input to the driver’s body model. The six motions of the moving platform are grouped into four modes as follows:

1. Longitudinal mode
2. Lateral mode
3. Heave mode
4. Yaw mode

The longitudinal mode contains surge and pitch as linear and angular motion components, respectively. Sway has linear motion and corresponding roll as an angular motion component in the case of the lateral mode. Heave mode only contains heave as a linear motion component and yaw is the only component in yaw mode. In each mode, the state-space equation can be obtained from the transfer function of the driver’s body models. The following text includes the description of the algorithm of longitudinal mode, where Ω_*p*_ is the angular velocity or derivative of pitch and *a*_*x*_ is linear acceleration along with surge.

The structure of the proposed MCA is given in Fig 6. Eq (22) shows longitudinal components of input *u*_*v*_(*s*) to simulator block.


$${\text{u}}_{\text{v}}(\text{s})=\left[\begin{array}{l} \Omega_{\text{p}}\\ {\text{a}}_{\text{x}} \end{array}\right]=\left[\begin{array}{l} {\text{u}}_{\text{v}1}\\ {\text{u}}_{\text{v}2} \end{array}\right].$$ (22)

The specific force *f*_*x*_(*s*) is defined as force per unit mass along surge and is given as follows:

$$f_{x}(s)=\left[\begin{array}{c} g\;\;\;0\\ 0\;\;\;\;1 \end{array}\right]\left[\begin{array}{c} u_{v1}/s\\ u_{v2} \end{array}\right].$$ (23)

The output of this cueing model is *u*_*s*_(*s*):

$$u_{s}(s)=\left[\begin{array}{l} {\hat{\Omega }}_{p}(s)\\ {\hat{f}}_{x}(s) \end{array}\right]=W_{s}(s)\left[\begin{array}{l} u_{v1}\\ u_{v2} \end{array}\right]=\left[\begin{array}{l} u_{s1}\\ u_{s2} \end{array}\right].$$ (24)

The problem is to determine a transfer function *W*_*s*_(*s*) that will be used to compute the output sensational signals for simulator moving platform position and orientation.

#### Determination of sensation signal

In optimal control theory, a cost function is to be optimized considering the motion system constraints [32–34]. In this case, the sensation error between virtual surroundings and real drivers is minimized. Sensation error can be determined by the observable canonical form of state space. In the case of angular motion channel, Eqs (25) and (26) are used, and in the case of translational motion channel, Eqs (27) and (28) are used.


$${\dot{X}}_{p}=A_{p}X_{p}+B_{p}u_{v},$$ (25)

$${\hat{\Omega }}_{p}=C_{p}X_{p}.$$ (26)

Similarly, using Eq (23),

$${\dot{X}}_{x}=A_{x}X_{x}+B_{x}u_{v},$$ (27)

$${\hat{f}}_{x}=C_{x}X_{x}.$$ (28)

Combining Eqs (25)–(28) with Eqs (29) and (30),

$${\dot{X}}_{v}=\left[\begin{array}{l} {\dot{X}}_{p}\\ {\dot{X}}_{x} \end{array}\right]=\left[\begin{array}{cc} A_{p} & 0_{9\times 16}\\ 0_{9\times 16} & A_{x} \end{array}\right]\left[\begin{array}{l} X_{p}\\ X_{x} \end{array}\right]+\left[\begin{array}{l} B_{p}\\ B_{x} \end{array}\right]u_{v}=A_{v}X_{v}+B_{v}u_{v},$$ (29)

$$\left[\begin{array}{c} {\hat{\Omega }}_{p}\\ {\hat{f}}_{x} \end{array}\right]=\left[\begin{array}{cc} C_{p} & 0\\ 0 & C_{x} \end{array}\right]\left[\begin{array}{l} X_{p}\\ X_{x} \end{array}\right].$$ (30)

If the input to driver’s body model is *u*_*s*_, then similar equations are obtained:

$${\dot{X}}_{v}=A_{v}X_{v}+B_{v}u_{s},$$ (31)

$$\left[\begin{array}{c} {\hat{\Omega }}_{p}\\ {\hat{f}}_{x} \end{array}\right]=C_{v}X_{v}.$$ (32)

Error dynamic equation can be obtained from Eqs (29)–(32) and is given as follows:

$${\dot{X}}_{e}=A_{v}X_{v}+B_{v}u_{s}-B_{v}u_{v},$$ (33)

$$e_{v}=C_{v}X_{e}.$$ (34)

It is seen in practical situations that the vehicle signal is accompanied by a filtered white noise signal, so it must be considered in the error signal. Eqs (35) and (36) show the state space of vehicle signal for noise considering w as filtered white noise input:

$${\dot{X}}_{n}=A_{n}X_{n}+B_{n}w,$$ (35)

$$u_{v}=X_{n}.$$ (36)

Adding the effect of white noise to the error dynamic equation for the determination of cumulative error *e*,

$$\dot{X}=\left[\begin{array}{l} {\dot{X}}_{e}\\ {\dot{X}}_{n} \end{array}\right]=AX+Bu+Hw,$$ (37)

e=CX, (38)

where

$$A_{n}=\left[\begin{array}{cc} -1 & 0\\ 0 & -4\pi \end{array}\right],B_{n}=\left[\begin{array}{l} 1\\ 1 \end{array}\right].$$

$$A=\left[\begin{array}{cc} A_{v} & -B_{v}\\ 0_{2\times 25} & A_{n} \end{array}\right],B=\left[\begin{array}{c} B_{v}\\ 0_{2\times 1} \end{array}\right],H=\left[\begin{array}{c} 0_{25\times 1}\\ B_{n} \end{array}\right],C=\left[\begin{array}{c} C_{v}\;\;\;0 \end{array}\right].$$

The main objective of this article is to produce realistic motion cues, i.e., motion cues similar to the real vehicle. To achieve this objective, the error between the vehicle signal and simulator motion signal, given by Eq (38), must be minimized. In the last section error, the dynamic equation is modeled; in this section, this error is minimized using the appropriate choice of the optimization method. As explained in the motion cueing section, there are workspace limitations of the motion simulator. These limitations must be included in the optimal control problem to generate realistic motion cues. Objective and constraints of motion system can be combined into a quadratic cost function and the problem is formulated as follows:

Find value of *u*_*s*_ that minimizes *J*:

$$J=\int_{t_{1}}^{t_{2}}\left(e^{T}Qe+u_{s}^{T}Ru_{s}\right)dt.$$ (39)

Subject to the following constraints,

$$l_{min}\leq l_{i}\leq l_{max},$$ (40)

$$v_{min}\leq v_{i}\leq v_{max},$$ (41)

$$a_{min}\leq a_{i}\leq a_{max},$$ (42)

$$X_{v}=\left[\begin{array}{c} {\hat{\Omega }}_{p}\\ {\hat{f}}_{x} \end{array}\right]=f\left(X_{p},u_{v}\right),$$ (43)

$$u_{s}=g\left(u_{v},w\right),$$ (44)

where *J* is the quadratic cost function to be minimized, *Q and R* are weighting matrices, and *l*_*i*_,*v*_*i*_,*a*_*i*_,*i* = 1,2,3,⋯6 are limb length, velocity, and acceleration of every leg of the motion system, respectively.

#### The enhanced sensitivity to the sensation error

The problem is to decide the inputs *u*_*s*_ such that our quadratic cost function is minimized within the limits of constraints. Fig 6 shows the input vehicle dynamics *u*_*v*_ as a vector of longitudinal acceleration, lateral acceleration, and yaw velocity. A similar vector *u*_*s*_ is generated as simulator input. Both of these inputs are used for the calculation of states of the system and outputs as discussed in Eq (30)–(38). The displacement, velocity and acceleration of the actuators are limited by the constraints of the cost function in Eq (40)–(42). The output error (*o*_*e*_) is the function of these states:

$$y(t)=\left[\begin{array}{c} a_{REF_{long\;}}(t)-a_{s_{long}}(t)\\ a_{REF_{lat}}(t)-a_{s_{lat}}(t)\\ v_{REF_{yaw}}(t)-v_{s_{yaw}}(t) \end{array}\right]$$ (45)

In short, there is a need of selecting such parameters which minimize the sensed longitudinal acceleration, lateral acceleration, and yaw velocity. The accelerations are translational whereas the velocity is a rotational parameter. However, this is a lengthy iterative process and requires repeated driver feedback. This work includes driver’s body model, to minimize the workload of drivers as shown in Eq (46) below:

$$y(t)=\left[\begin{array}{c} a_{REF_{long}}(t)-a_{s_{long}}(t)-a_{v_{long}}(t)\\ a_{REF_{lat}}(t)-a_{s_{lat}}(t)-a_{v_{lat}}(t)\\ v_{REF{\;}_{yaw}}(t)-v_{s_{yaw}}(t)-v_{v_{yaw}}(t) \end{array}\right]$$ (46)

#### Analysis of cost function

We require to cue the brake system and the accelerate system while in longitudinal mode. For both situations we use numerical control techniques for error free acceleration profiling. The basicentric coordinate system of a human body in Fig 5 shows the direction of the longitudinal acceleration, lateral acceleration, and yaw velocity.

**Fig 7:**
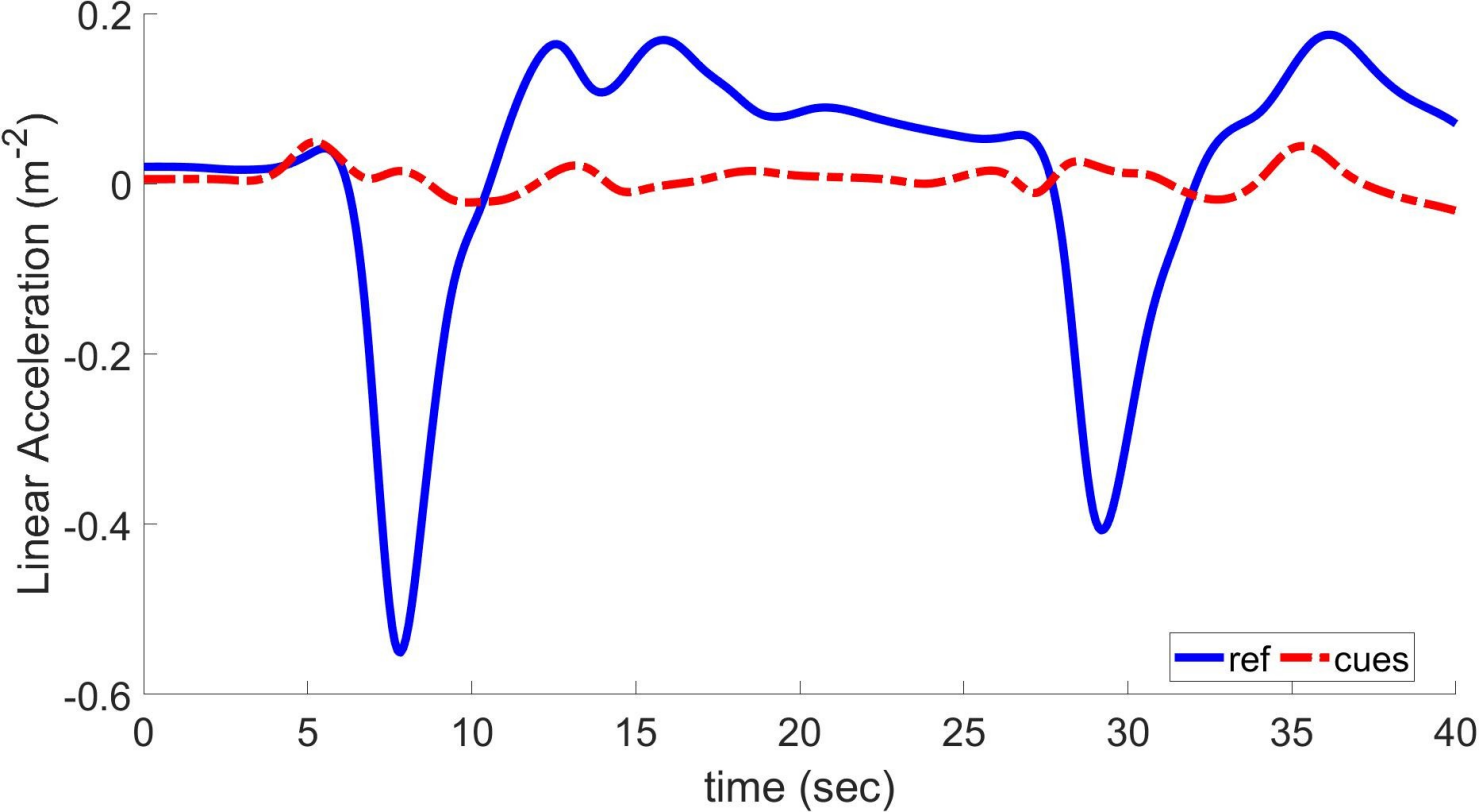
Longitudinal acceleration and resulting cues using linear quadratic regulator based on optimal MCA.

The reference (blue solid line) and the simulated (red dashed line) linear acceleration in Fig 7. The simulated wave is obtained by minimizing the time invariant objective function from Eq (44) with only minimizing the square of the sensation error. The platform must start the maneuver at the front of the workspace and must conclude it there for the optimum reaction to operate. As a result, the platform was set up for braking, and the maneuver concluded in an acceleration-ready posture. This cueing signal has two characteristics that need discussion. The first is a delayed reaction that causes the platform’s peak deceleration to coincide with the cars. The platform comes to a stop near the end of the braking action due to a mistake made during workspace management, which is the motion’s second distinguishing feature. This answer falls short of what the racing car drivers we contacted demand in terms of a strong early onset cue, hence it is not acceptable. Discussions led to the conclusion that drivers need a strong first onset cue followed by a mistake of the smallest feasible size. Since the cueing acceleration produced lacks these traits, it needed to be modified.

In a drawn-out high-speed turn, lateral acceleration signals can be of great amplitude and extended duration and have a substantially different form from the longitudinal case. Simulators with a constrained workspace cannot simulate this kind of acceleration. Again, the acceleration at the beginning is cued but not maintained. Since the yaw acceleration and the lateral cue have a similar shape, an objective function of the type Eq (44) is also utilized in this situation.

## Results and discussion

A good motion cueing system should possess the following properties:

1. Realistic motion cues, i.e., the wave shape of output acceleration cues, should be more similar to that of input linear acceleration. Similarly, the wave shape of output angular velocity cues should be more similar to that of input angular velocity.
2. Usage of more workspace, i.e., the limits of theoretical workspace, should be achieved by optimum position and orientation of motion base.
3. It does not generate false cues.

In this article, all the above three qualities have been practically achieved. Results are compared based on the root-mean-square difference (RMSD) and coefficient of cross-correlation (COCC) that show similarity between two waveforms to produce realistic motion cues. The following section includes the application of control strategies step by step for motion cueing and compares the results.

### State-of-the-art motion cueing algorithms

Optimal MCA based on the linear quadratic regulator (LQR) is modified by changing the axis of rotation to the driver’s body axis instead of the motion system’s center of rotation [16, 22]. The main aim behind this change is to achieve realistic motion cues and remove false cues. Fig 5 shows the basicentric coordinate system of the driver body (seated position). The cross-coupling between the simulator and driver depends on the distance between their respective coordinate systems. As discussed in the motion cueing section, MCA receives linear acceleration and angular velocity as input to produce a proper cueing signal for the simulator motion base. Furthermore, a tilt rate limiter is introduced after cueing has been achieved for better tilt coordination. This addition investigates the effects on both motion cue fidelity and displacement.

**Fig 8:**
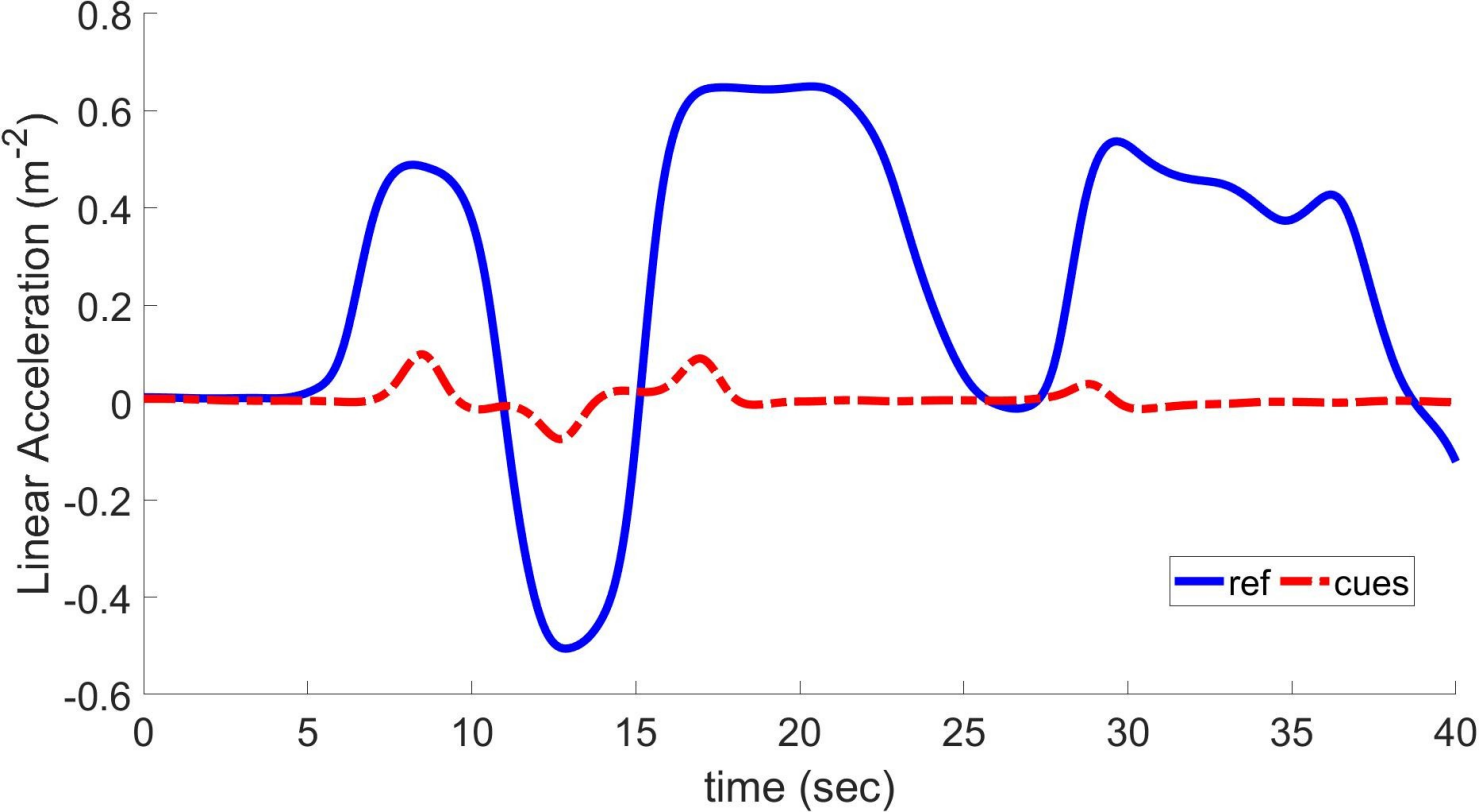
Lateral acceleration and resulting cues using linear quadratic regulator based on optimal MCA.

The translational input has time values of longitudinal, lateral, and heave-specific forces. These inputs vary depending on acceleration, deceleration, and retardation, as shown in Figs 7 and 8. In these Figs., the blue plot shows values of input linear acceleration and illustrates that these values are never uniform; i.e., they keep on changing with time. According to Fig 7, longitudinal specific force values are applied to linear quadratic regulator-based optimal MCA, and resultant cues are plotted in a red dashed line. By thorough investigation of this plot, it can be observed that the coherence between waveforms is less guaranteed when specific force is changed quickly. Therefore, the LQR-based MCA can better track and follow the input signals during uniform conditions. The NOC-based technique [17] has a poor COCC of 0.8 and more RMSD of 0.455 than the LQR-based method, which has an improved COCC of 0.88 and minimized RMSD to 0.218. This shows that the LQR method provides much better results in the form of generating realistic motion cues. RMSD should be decreased to zero to achieve realistic motion cues.

Fig 8. shows the comparison between normalized lateral specific force inputs and generated motion cues using the LQR-based optimal MCA. Like the previous case of longitudinal specific force, it is observed that the coherence between waveforms is less guaranteed when the specific force is changed quickly. Therefore, LQR-based MCA can better track and follow the input signals during uniform conditions. The NOC-based technique [17] has a lower COCC of 0.83 and RMSD of 0.196 than the LQR-based method, which has an improved COCC of 0.86 and increased RMSD to 0.221. This shows that the LQR method provides much better results in the form of generating realistic motion cues. Greater RMSD shows the doubtful performance of the algorithm during sudden changes of input like braking. To achieve realistic motion, a more precise MCA must be developed that minimizes RMSD to zero.

**Fig 9:**
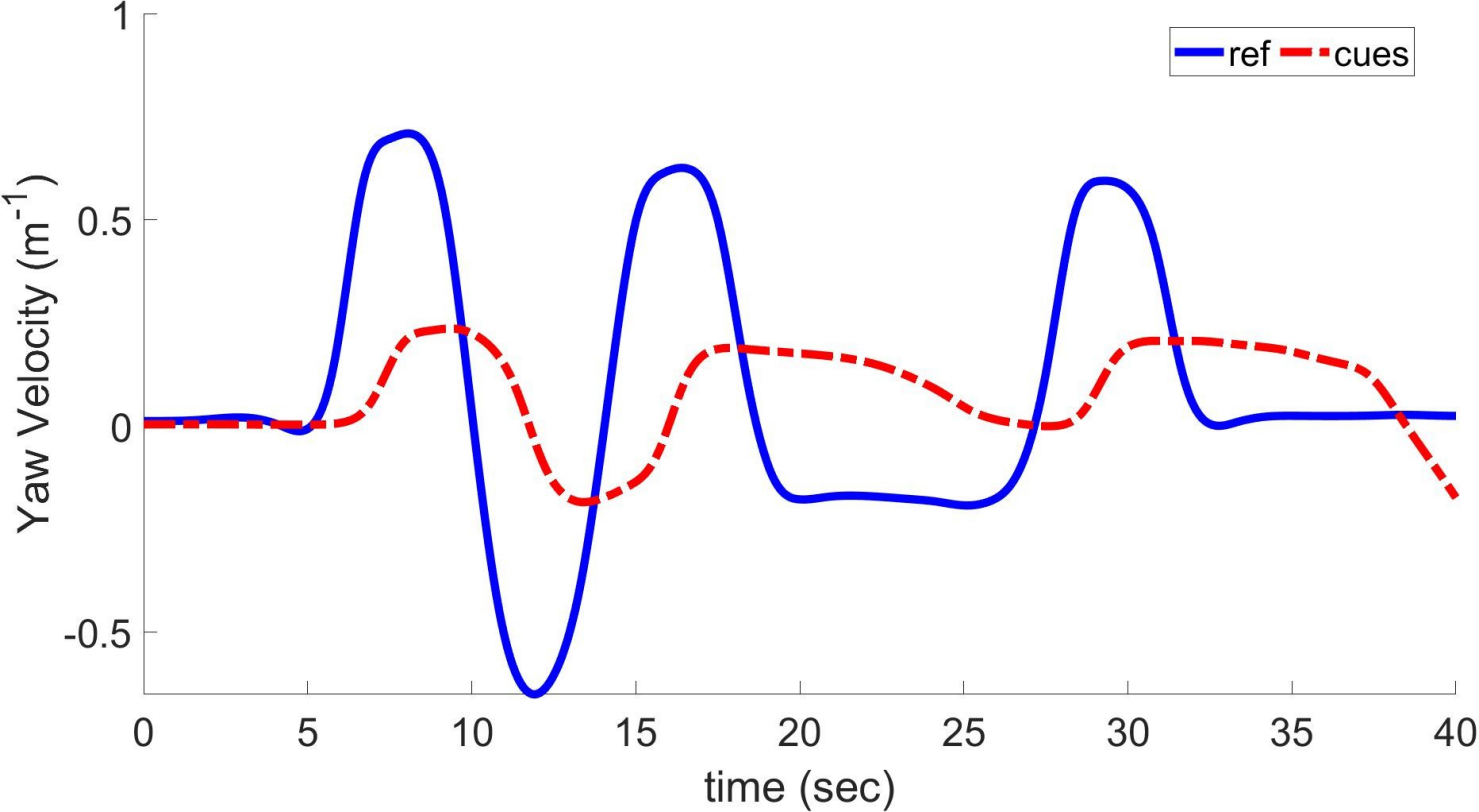
Yaw velocity and resulting cues using linear quadratic regulator based on optimal MCA.

Fig 9 shows the comparison between yaw velocity and generated velocity cues based on the LQR-based optimal MCA. In contrast to the numerical optimal MCA, this method has an improved COCC of 0.89 and an angle of 3.22. The RMSD was minimized to 0.219. In the case of NOC, these values are 0.81, 2.23, and 0.417, respectively. This shows that LQR-based MCA can decrease sensation error between virtual surroundings and real drivers. The improvement is significant compared with the previous MCA, but it still needs to be improved.

We now compare two popular motion cueing methods based on LQR and GA. The LQR method discussed earlier in this section shows some improvements in RMSD and COCC when compared with the NOC strategy, but it still has undesirable RMSD. All methods are based on the mathematics derived in the motion cueing section, which employs human perception and the human body model to achieve desired results.

These methodologies improve sensation signals by minimizing RMSD and improving the COCC. Using more simulator workspace results in increased sensation error, but an optimal solution may result in the optimized workspace with RMSD as minimum as possible, which needs to have more constraints to be considered with relative simplicity in each constraint.

For GA implementation, some parameters have to be chosen, for example, population size, crossover rate, and mutation rate. By choosing these parameters, the selection is made between the degree of freedom of solution and speed of convergence of the GA. Thus, proper selection of crossover and mutation rate should be ensured to obtain the best results [16]. Depending on different applications, these parameters may vary as they are chosen by trial and error. In the case of a large mutation rate, the individual chromosomes may jump to the closer solution, whereas in the case of a small mutation rate, they may stick in local minima.

The recommended settings of these parameters are included in [22]. GA consists of the settings of crossover rates and mutation rates, which are satisfactory and meet the demands of the experimental study. Evolutionary operators are assigned to individual chromosomes that constitute the population. It should be noted that the search capacity of the GA is limited because of the small number of chromosomes in the population. If the number of chromosomes increases, the computational time also increases, not suitable for the proposed application. Therefore, an optimal value for population size should be selected to avoid the slow performance of the algorithm. From [7], the population size of fifty chromosomes is appropriate for acceptable results. If the number of chromosomes increases, it will not improve the solution considerably; however, it will significantly decrease computational speed and increase the time of convergence of the solution.

Alternatively, chromosomes are initialized based on the values of the LQR method, as discussed in the previous section. According to the literature [7], the parameter settings for GA parameters are as follows: the crossover rate is 0.8, the mutation rate is 0.2, and half of the size of the chromosomes for individual regeneration.

As the population size tends to increase, the results become more satisfactory in terms of sensation error, but the computational speed tends to decrease accordingly. According to a convergence test on 20, 50, and 100 chromosomes in [16], the best fitness function value, human sensation error, and displacement are obtained using a population size of 50 chromosomes instead of 20. No fruitful results are achieved by increasing the population size from 50 chromosomes. Finally, GA has been successful in decreasing human sensation error and displacement and improving the COCC.

It must be kept in mind that the speed of computation for a population size of 100 chromosomes decreases significantly, resulting in an increased computational burden. When the population size is selected greater than 50, the speed of computation becomes too slow without significant improvement in fitness function value.

Computational time per iteration in the case of 20 chromosomes is 13.90s, whereas in the case of 50 chromosomes is 27.72 s and in the case of 100 chromosomes, it is 52.06 s [16]. GA-based optimal MCA stops searching at 455th iteration in case of 50 chromosomes. The density of genetic problems is based on several constraints, generations, and iterations per second, tends to increase with the increase in population size, and is less significantly affected using a population size of 50 compared to a population size of 100.

**Fig 10:**
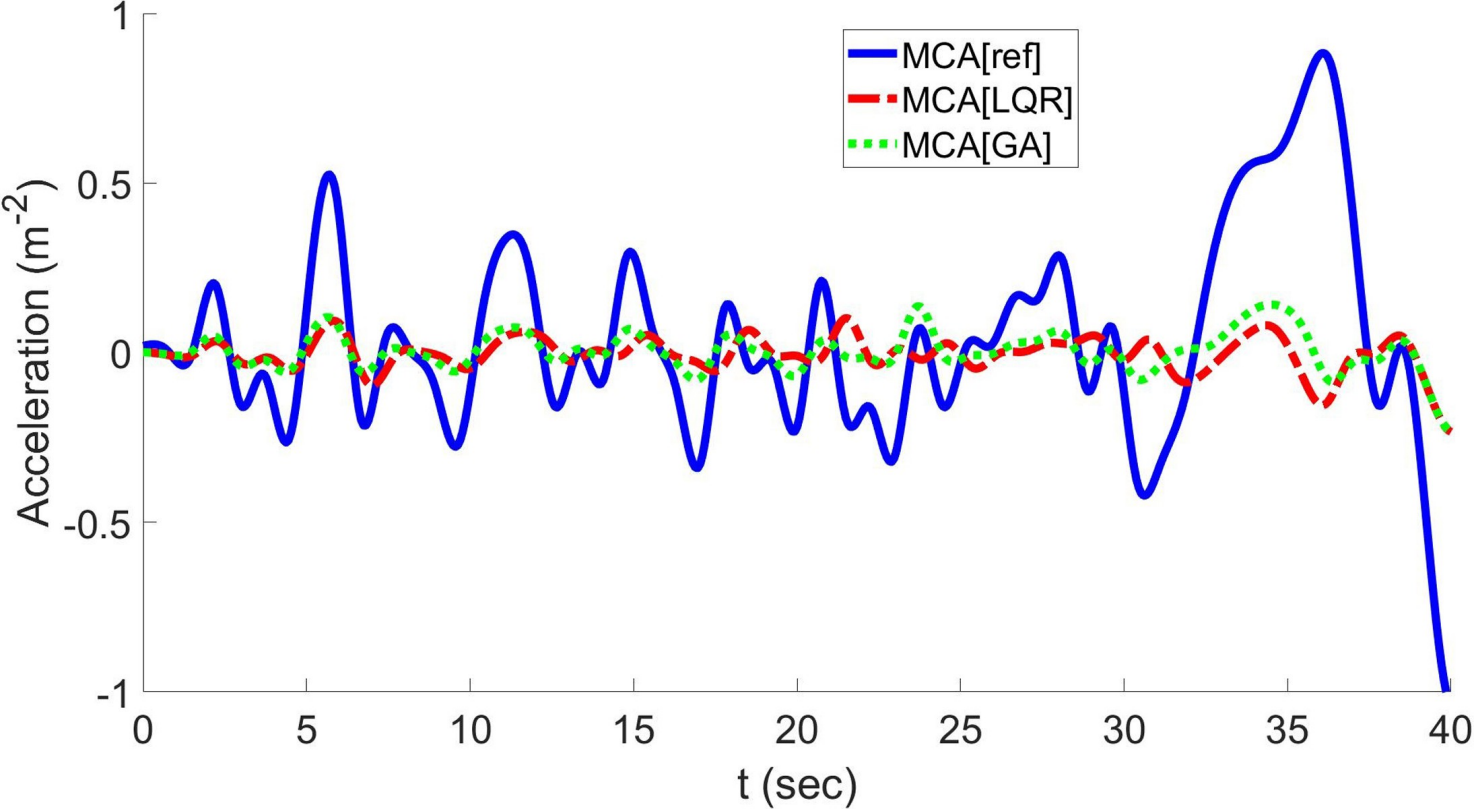
Plots of longitudinal acceleration and resulting cues show a comparison between the MCAs based on MPC, LQR MCA, GA, and the proposed one.

Fig 10 shows normalized values of reference longitudinal linear accelerations for comparing MPC-, LQR- and GA-based optimal MCAs with the proposed one. A range of longitudinal acceleration, deceleration, and retardation is applied to the LQR-based optimal MCA and GA-based optimal MCA. The resulting longitudinal acceleration cues are generated and plotted in Fig 10.

According to Fig 10, the GA-based optimal MCA shows better performance than the LQR method because the GA can track input-specific force with high accuracy. In addition, false cues are removed using the GA.

A comparison of RMSD and COCC between various algorithms is shown in Tables 1 and 2, respectively. The RMSD has been compared with NOC [17], LQR [16], GA [33], OPT [35], and MPC [36]. From the Table 1 is can be seen that the NOC behaves imperfectly in case of longitudinal acceleration and yaw velocity, whereas lateral acceleration is inefficiently in case of MPC. This is because the NOC is more related to conventional techniques and MPC cannot better predict the model while in lateral direction. The proposed technique is well suited in all cases as it employs the extended sensation error. Similarly, while we see the coefficient of cross correlation in Table 2 for the sake of comparison between the above techniques, our results are comparable to the GA based approach in all cases of longitudinal & lateral acceleration and also in yaw velocity. This is because our methodology is more closely related to the OPT and GA, benefitting from both. Later we also plot these values of COCC in Fig 10 for the sake of visual comparison with MPC, LQR, GA based techniques.

**Table 1:** Comparison of RMSD.

	*A*_*long*_	*α*_*lat*_	*v*_*yaw*_
**NOC [17]**	0.455	0.196	0.417
**LQR [16]**	0.218	0.221	0.219
**GA [33]**	0.072	0.092	0.082
**OPT [35]**	0.335	0.271	-
**MPC [36]**	0.194	0.310	-
**Proposed**	**0.022**	**0.036**	**0.013**

**Table 2:** Comparison of COCC.

	*A*_*long*_	*α*_*lat*_	*v*_*yaw*_
**NOC [17]**	0.80	0.83	0.81
**LQR [16]**	0.88	0.86	0.89
**GA [33]**	0.95	0.96	0.98
**OPT [35]**	0.90	0.92	-
**MPC [36]**	0.68	-	-
**Proposed**	**0.98**	**0.97**	**0.98**

According to Fig 10, the sensation error between virtual surroundings and the real driver has decreased in the case of the GA method compared to the LQR method. This error can be further decreased and taken below the human threshold using other techniques such as one utilized in the next subsection using H∞ control.

A comparison of maximum displacement and maximum angle between various algorithms is shown in Tables 3 and 4, respectively. As shown in Table 3, it is clear that displacement of the simulator in surge direction using GA-based optimal MCA significantly drops to 0.37, which was 0.44 in the case of LQR-based optimal MCA. However, the OPT based approach results least values of both surge and Heave. This can be due to the constraints applied by their methodology. Moreover, the yaw angle from Table 4 in the case of GA-based optimal MCA decreases to 2.04, which was 3.22 in the case of LQR-based optimal MCA. Hence, the GA-based optimal MCA reduces workspace usage by decreasing RMSD and improving COCC.

**Table 3:** Comparison of maximum displacement.

	Surge (m)	Sway (m)	Heave (m)
**NOC [17]**	0.33	0.37	0.20
**LQR [16]**	0.44	0.45	0.21
**GA [33]**	0.37	0.39	0.18
**OPT [35]**	0.27	-	0.18
**Proposed**	**0.50**	**0.49**	**0.22**

**Table 4:** Comparison of maximum angle.

	Roll (^*o*^)	Pitch (^*o*^)	Yaw (^*o*^)
**NOC [17]**	2.50	2.79	2.23
**LQR [16]**	3.50	2.98	3.22
**GA [33]**	0.37	0.39	0.18
**Proposed**	**3.33**	**4.34**	**3.58**

### **H**∞ **control-based optimal MCA**

The human body model and human perception model have been integrated into the classical washout filter to observe the sensation error, the error between virtual surroundings and real driver. Driver’s health comfort and motion sickness are significantly apparent if this error is more than a specified threshold. To minimize this error, hence, to produce realistic motion cues, optimal motion cueing is applied using different optimization methods, like LQR, GA, PSO, and H∞.

All previous methods result in some acceptable improvement in error with less workspace usage. H∞ control strategies are helpful for their ready applicability to systems involving multiple variables with cross-coupling between channels. However, for an H∞ controller, it is difficult to optimize robust performance and stabilization simultaneously. However, many methods, known as extensions of this strategy, exist, such as H∞ loop-shaping, which permits the designer to apply classical loop-shaping concepts to the frequency response of the multivariable system and achieve robust performance.

Then favorable robust stabilization is achieved by optimizing the system’s response near bandwidth frequency. Here, the Riccati equation is reformulated using optimization and linear matrix inequalities with fewer constraints.

**Fig 11:**
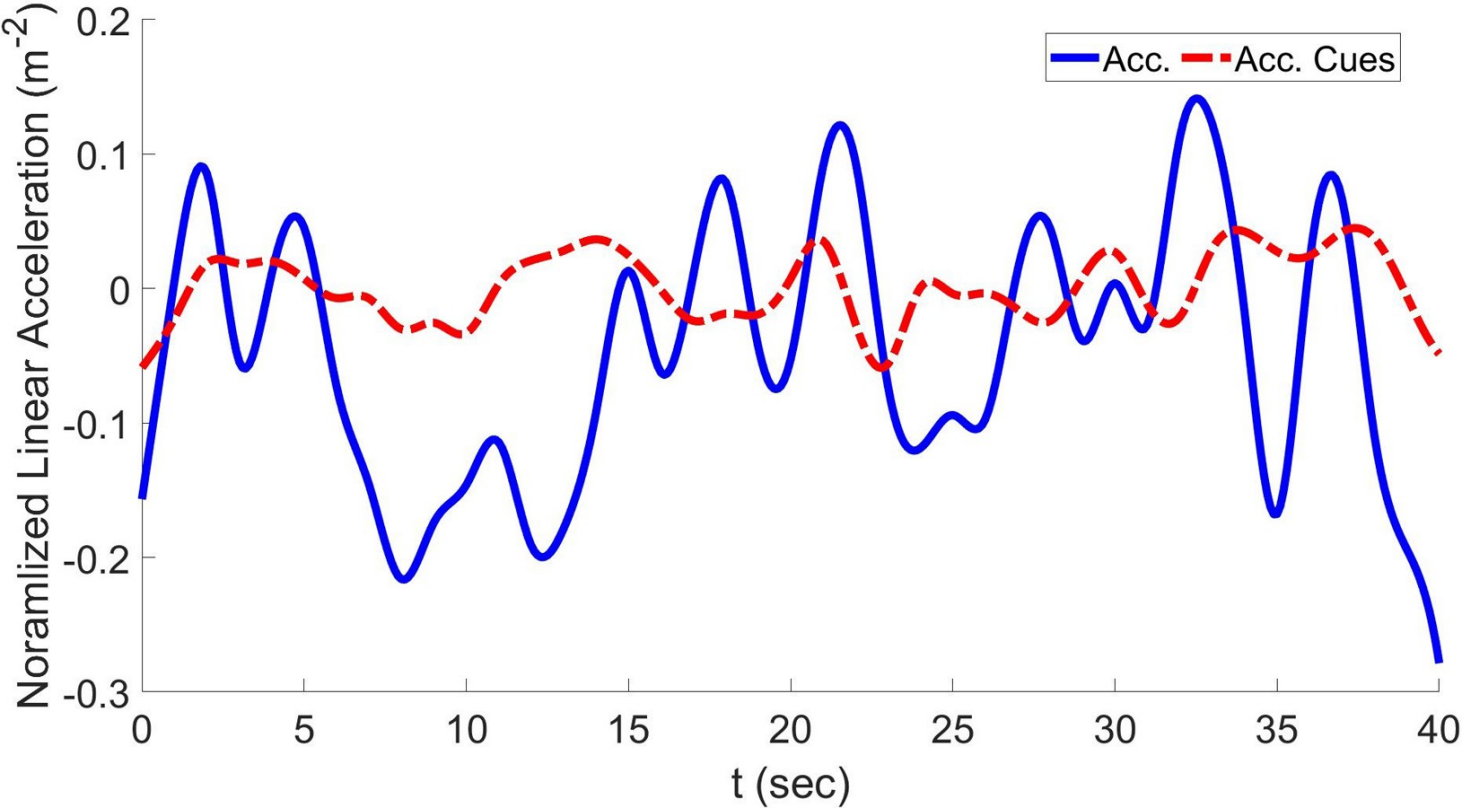
Lateral acceleration and resulting cues using the proposed approach.

**Fig 12:**
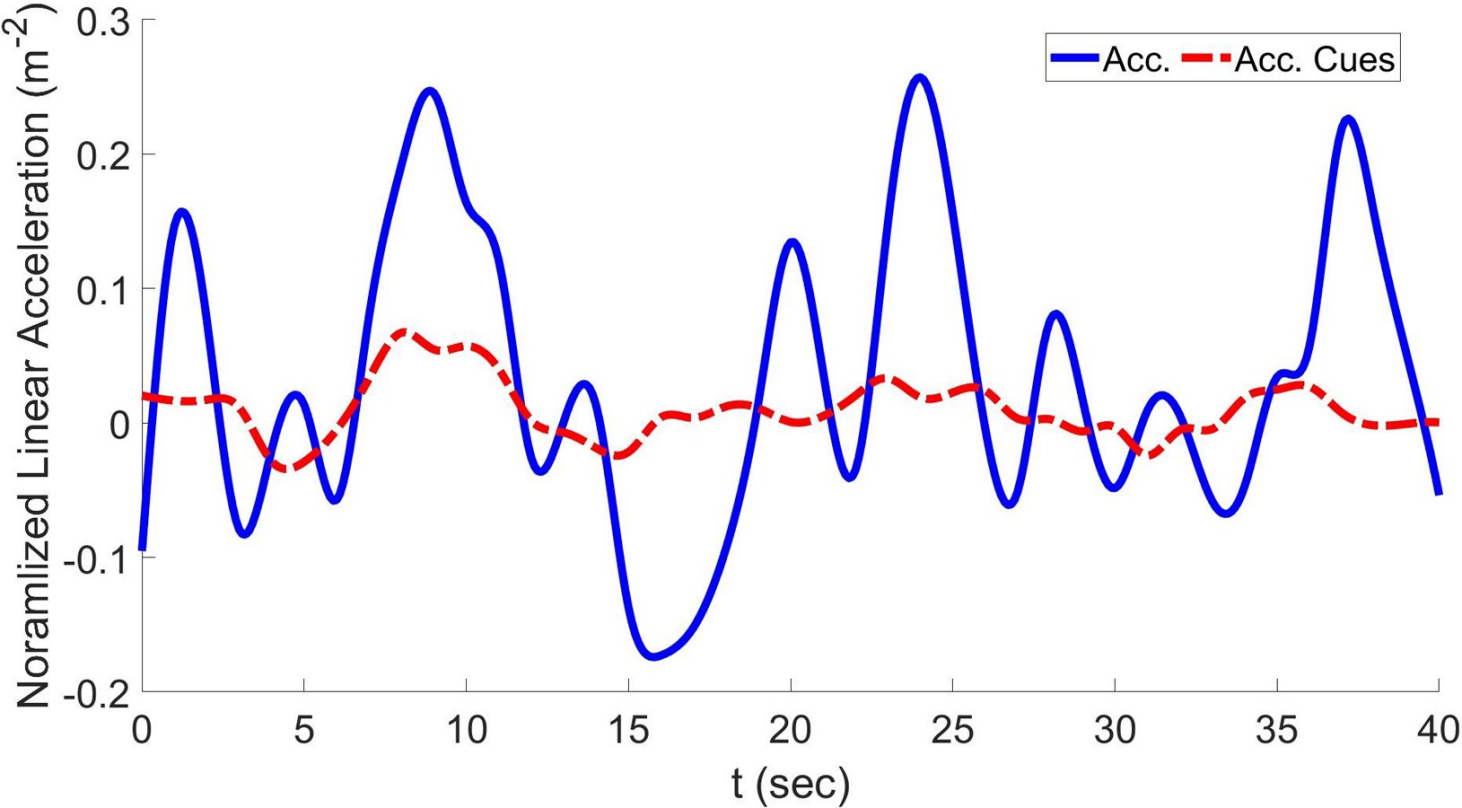
Longitudinal acceleration and resulting acceleration cues using the proposed approach.

**Fig 13:**
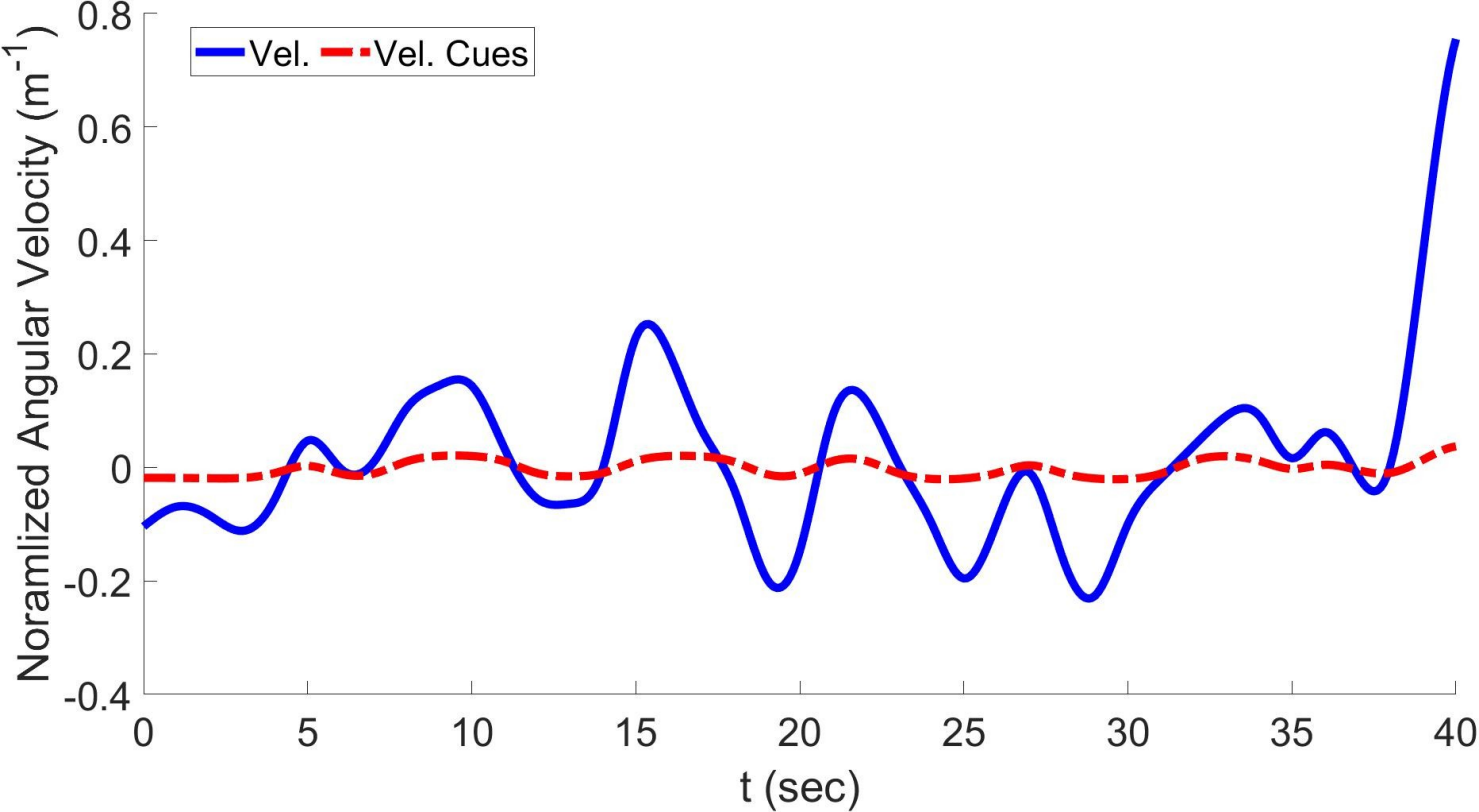
Yaw velocity and resulting cues using the proposed approach.

Figs 11–13 show the results of H∞ control-based optimal MCA in the case of lateral accelerations, longitudinal acceleration, and yaw velocities, respectively, compared to reference signals along with their cues.

The sensation error between the simulator and driver and the time derivative of the sensational reference signal is input to the H∞ control method. Then, the H∞ controller generates a supplementary reimbursing signal “Fi,” which removes the sensation error between the simulator and driver. The human body has limited ability to enumerate motion applied to it, and the human vestibular system cannot differentiate between small, medium, or large motions in various directions. This motion is divided into logical perceptible ranges. Therefore, in this proposed H∞ control method, sensation error values have been divided into five groups: heavily positive, positive, zero, negative, and heavily negative. In this way workspace of the simulator is improved.

In the translational channel of the H∞ controller, both inputs are classified as the otolith organ and vestibular organ. The result of the high-pass filter is input to this block and then compared with vehicle input. Then, this error is compared as an input to H∞ controller that eliminates error in translational sensational signal.

In the rotational channel of the H∞ controller, inputs are provided through the otolith organ and vestibular organ. The result of the low-pass filter is input to this block for tilt coordination and then compared with linear vehicle input to determine the error. Then, this error is then provided as an input to the H∞ controller.

As seen in Table 1, in the case of GA-based MCA, the RMSD in the longitudinal direction is 0.072 and the COCC is 0.95. In the case of H∞-based optimal MCA, RMSD is significantly reduced to 0.022 and the COCC is significantly improved to 0.98. Moreover, in the case of GA-based optimal MCA, the RMSD in the lateral direction is 0.092 and the COCC in the same direction is 0.96. In the case of the H∞-based optimal MCA, RMSD is significantly reduced to 0.036 and the COCC is significantly improved to 0.97. The RMSD in the yaw velocity direction in the case of GA-based optimal MCA is 0. 082 and the COCC in the same direction is 0.98. In the case of the H∞-based optimal MCA, RMSD is significantly reduced to 0.013 and the COCC is significantly improved to 0.98.

The RMSD, COCC, maximum displacement, and maximum angle of NOC, LQR, GA, and H∞ control-based MCAs are given in Tables 1–4, respectively.

From Table 3, H∞-based optimal MCA shows a significant increase in displacement of the simulator to 0.50 in the surge direction. In the case of LQR-based optimal MCA, it is 0.44; in the case of GA-based optimal MCA, it is 0.37. Also, the yaw angle is increased to 4.34 in the case of H-based optimal MCA, 3.22 in the case of LQR-based optimal MCA, and 2.04 in the case of GA-based MCA. Hence, the H∞-based optimal MCA improves workspace usage by decreasing RMSD and improving the COCC. This leads to effective implementation of the H∞-based optimal routine for motion cueing of driving simulators, considering fewer constraints, and resulting in more workspace usage.

The proposed MCA confirms the production of realistic motion cues by minimizing RMSD. Workspace usage is maximized in two ways:

1. Optimally solving forward kinematics
2. Using H∞ controller

This leads to the effective implementation of optimal routine for motion cueing of driving simulators, considering fewer constraints and resulting in more workspace usage.

In short, all the current optimal MCAs do not utilize a sufficient simulator workspace. Linear data fitting techniques for motion cueing problems are limited by second-order performance indices, such as acceleration values. In contrast, model predictive control involves different systems with different physical constraints. NOC overcomes these limitations by integrating time-varying cost functions adjusted by driver’s feedback. Although NOC is an open-loop method, it is still helpful in analyzing performance constraints in loop strategies. Other methods, such as LQR-based optimal control and GA-based optimal control, show better performance in improving the sensation signal. However, they cannot enhance workspace usage due to more constraints involved. The LQR-based method improves the workspace but has more sensation error, and the GA-based method decreases error but with poor workspace usage.

## Conclusion

This research proposes a novel optimal MCA for vehicle driving simulators that decreases the sensation error between virtual surroundings and real drivers. The quadratic cost function of the error between vehicle signal and simulator output is minimized using the conventional H∞-based optimal control. In addition, usage of the simulator workspace is improved by implementing driver’s body model with driver’s perception model in optimal MCA. The procedure to design optimal MCA *W*(*s*) and the performance of this algorithm through computer simulations have been presented. The simulation results have shown significant improvement both quantitatively and qualitatively.

For future work, other optimization techniques like PSO can be applied for assessment. Moreover, fuzzy logic controller-based optimal MCA can be employed. Like the H∞ controller, a fuzzy logic controller can be adopted to enhance motion fidelity.

## Abbreviations

*α_lat_*: Lateral acceleration
*α_long_*: Longitudinal acceleration
* $\hat{s}$ *: Orientation of moving platform in screw axis notation
*θ*: Angle of screw axis in screw axis notation
${\hat{f}}_{x(s)}$: Sensation signal in case of linear motion
*^A^P*: Position of moving platform
*^A^a_i_*: Position of *A*_*i*_ with respect to *O*_*A*_
*^A^b_i_*: Position of *B*_*i*_ with respect to *O*_*A*_
*^B^b_i_*: Position of *B*_*i*_ with respect to *O*_*B*_
*A_i_*: *i*^*th*^ spherical joint at the base platform of Stewart platform
*a_x_*: Linear acceleration along the surge
*B_i_*: *i*^*th*^ spherical joint at moving platform of Stewart platform
*d_k_*: Search direction identifier in Gauss–Newton method
*dof*: Degrees of freedom of robot
*e*: Error between sensational signal and vehicle signal
*f*_*x*_(*s*): Force per unit mass along surge
*G_s_*: Transfer function for acceleration transition
*G_T_*: Transfer function for velocity transition
*G_Ω_*: Ratio of vestibular organ to rotational velocity
*G*_*ρ*_: Ratio of otolith organ to the specific force
*G_BL_*: Band-limiting filter
*G_HP_*: High-pass filter
*G_LP_*: Low-pass filter
*J*: Quadratic cost function
*j*: No. of joints of a robot
*L*: Vector of given limb lengths
*l_i_*: *i*^*th*^ limb length of *i*^*th*^ kinematic chain of Stewart platform
*n_L_*: No. of links of a robot
*O_A_*: Coordinate frame attached to base platform
*O_B_*: Coordinate frame attached to moving platform
*P_i_*: *i*^*th*^ prismatic joint between fixed and moving platform
*p_x_*: *x*-coordinate of position of moving platform
*p_y_*: *y*-coordinate of position of moving platform
*p_z_*: *z*-coordinate of position of moving platform
*Q*: Weighting matrix for error *e*
*R*: Weighting matrix for input signal *u*_*s*_
*t*: Vector of position and orientation in screw axis notation
*u*_*s*_ (*s*): Input to driver’s body model
*u*_*v*_ (*s*): Input to simulator
*v_yaw_*: Yaw velocity
*W*(*s*): Transfer function for optimal motion cueing
*λ_*k*_*: Search direction identifier in Levenberg–Marquardt Method
*Ω_*p*_*: Angular velocity or derivative of pitch
${\hat{\Omega }}_{p}(s)$: Sensation signal in case of angular motion
*dof_i_*: Degrees of freedom of *i*^*th*^ joint
*dof_p_*: Degrees of freedom of passive mechanism
*dof_s_*: Degrees of freedom of space
${}_{B}^{A}R$: Orientation of moving platform in rotation matrix notation
